# Metformin induces a prompt decrease in LH-stimulated testosterone response in women with PCOS independent of its insulin-sensitizing effects

**DOI:** 10.1186/1477-7827-12-98

**Published:** 2014-10-11

**Authors:** Dorothea Kurzthaler, Dijana Hadziomerovic-Pekic, Ludwig Wildt, Beata E Seeber

**Affiliations:** Department of Gynecologic Endocrinology and Reproductive Medicine, Medical University of Innsbruck, Anichstrasse 35, A-6020 Innsbruck, Austria; Kinderwunsch Zentrum Goldenes Kreuz, Lazarettgasse 16, A-1090 Vienna, Austria

**Keywords:** PCOS, Insulin-sensitizing drugs, Androgens, Testosterone

## Abstract

**Background:**

The use of insulin-sensitizing drugs has been shown to improve both the reproductive and the metabolic aspects of PCOS. However, the mechanisms by which metformin exerts its effects in PCOS are still not completely understood. There is growing evidence of a direct effect of metformin on ovarian steroidogenesis, independent of its effects on insulin sensitivity.

**Methods:**

We evaluated the short-term effects of metformin compared to placebo on basal and LH- stimulated androgen secretion as well as on hormonal and metabolic parameters in 19 women with PCOS during a four-day randomized, double-blinded placebo-controlled clinical trial. In a three month follow-up evaluation, we investigated the longer-term therapeutic effects of metformin on ovulation, metabolic and endocrine parameters.

**Results:**

Compared to placebo, 2 days of metformin was associated with a borderline significant reduction in the free androgen index (FAI) (p = 0.05) and with a reduction in the serum concentration of LH-stimulated testosterone (T) (p = 0.03). Following three months of use, a decline in serum T was observed, independent of changes in weight, metabolic parameters, or insulin sensitivity.

**Conclusions:**

In women with PCOS, Metformin induces a prompt decrease in LH-stimulated T secretion after only several days of use. This action precedes the medication’s effects on insulin sensitivity or weight loss.

## Background

Polycystic ovary syndrome (PCOS) is characterized by elevated circulating androgen levels and/or clinical signs of hyperandrogenism, chronic oligo- or anovulation and the presence of polycystic ovaries in ultrasound [1]. Additionally, women with PCOS often have profound insulin resistance and an alteration in pancreatic β-cell function [2, 3].

The mechanism by which metformin exerts its effects in PCOS is still not completely clear. Some [4–6], but not all clinical studies have shown an improvement in insulin sensitivity with metformin use [7, 8]. In vitro studies using cultured ovarian cells have demonstrated a direct metformin-effect on ovarian steroidogenesis [9].

The effects of metformin on adrenal steroidogenesis have been evaluated in several studies. La Marca et al. suggested that a one-month course of metformin therapy (1500 mg/day) reduces plasma concentrations of free testosterone and the adrenal steroidogenic response to ACTH [10]. However, a study using 1000 mg/d for a longer period of 12 weeks did not corroborate these findings [11].

Studies have shown that women with PCOS exhibit increased expression of cytochrome P450c17α (CYP 17), which results in increased activity of 17α-hydroxylase and 17,20-lyase in ovarian theca cells [12, 13]. This dysregulation of CYP 17 enhances the production of 17α-hydroxyprogesterone, androstenedione and testosterone. In cultured theca cells from women with PCOS, insulin significantly increased the production of androstenedione after luteinizing hormone (LH) stimulation [14]. Clinically, decreases in ovarian cytochrome P450c17α activity and serum free T have been shown following metformin therapy, thought to be secondary to a reduction in insulin secretion [15].

However, it has likewise been shown that insulin-sensitizing agents used for the treatment of PCOS exhibit insulin-independent ovarian effects [16]. For example, the thiazolidinediones directly inhibit ovarian androgen production in human ovarian cells in the absence of insulin [17]. Most previously published studies reporting improvement in hyperandrogenemia have evaluated androgen levels in PCOS women following 8 to 12 weeks of metformin therapy [4, 7], though some have observed a reduction in testosterone within 1 week of commencing treatment [18]. Few studies have tested insulin sensitivity dynamically with an oral glucose tolerance test performed pre- and post-treatment, making it difficult to delineate whether improvements in hyperandrogenemia were a direct effect of metformin or a secondary effect due to decreased insulin concentrations.

The primary aim of this study was to test the hypothesis that metformin may temper the typical hyperandrogenic ovarian response seen in PCOS by a direct effect on the enzymatic system of the ovary, independent of its action of improving insulin sensitivity in women with PCOS. The secondary aim of the study was to determine the longer-term effects of metformin on weight, menstrual cyclicity, metabolic and endocrine parameters following a twelve week course of treatment.

## Methods

### Study design and subjects

The study protocol was approved by the Ethics Committee of the Innsbruck Medical University and written informed consent was obtained from all participants. The study was registered with the European Union Drug Regulating Authorities Clinical Trials (EudraCT) with the identifier EudraCT 2006-001911-31.

This was a randomized, double-blinded, placebo-controlled clinical trial performed over four days and followed by a twelve week follow-up period. The primary outcome was short-term change in androgen levels and stimulated androgen response to LH following 2 days of medication use. Secondary outcomes included changes in anthropometric measures, insulin sensitivity, ovulation rate, menstrual frequency, and metabolic and endocrine parameters during the twelve week follow-up period.

Women with PCOS were recruited from the Department of Gynecological Endocrinology and Reproductive Medicine of the Innsbruck Medical University (Innsbruck, Austria). To be eligible for the study, subjects had to be between 18 and 40 years of age and have PCOS, as defined by the Rotterdam consensus criteria [1]. Namely, the women had to have at least two out of three of the following: (1) menstrual disorders defined as oligomenorrhea (cycle length > 35 d) or amenorrhea (cycle length > 12 weeks), (2) clinical hyperandrogenism (modified Ferriman-Gallwey score of ≥ 6, presence of acne or seborrhea) and/or biochemical evidence of hyperandrogenemia (T > 0.4 ng/ml, an established and validated cut-off at our university), and/or (3) polycystic ovaries by ultrasound.

Exclusion criteria included: diabetes mellitus, hyperprolactinemia, thyroid disorders, late-onset congenital adrenal hyperplasia, Cushing’s syndrome and liver or kidney diseases, current pregnancy, history of lactic acidosis, and the use of medications such as insulin sensitizers, insulin, anti-epileptics, H2-blockers, cholesterol-lowering drugs and diuretics. As such, all participants underwent a hormonal profile, an oral glucose tolerance test and an ACTH-stimulation test to exclude secondary causes of hyperandrogenism and manifest diabetes.

None of the participants had taken hormonal contraceptives, medications that affect gastrointestinal motility or carbohydrate metabolism for at least 2 months prior to study begin.

### Study protocol

We recorded the results of a routinely performed 75 g oral glucose tolerance test with glucose and insulin levels measured at time 0 (fasting, before the glucose load), and every 15 minutes over a total time of 3 hours. This test was performed in all participants within the previous 30 days prior to beginning the study.

The study was undertaken in the early follicular phase following spontaneous or progesterone-induced menstruation (cycle day 3–5). On study day 1, all women underwent clinical assessment (age, weight, height, BMI, blood pressure) and peripheral venipuncture to assess serum hormonal concentrations of estradiol (E2), Progesterone (P), FSH, and LH to confirm cycle phase.

Immediately following the baseline assessment, an LH stimulation test was performed with a load of 75 I.E. recombinant human LH (Lutropin alfa, Luveris®, Merck Serono, Aubonne, Switzerland) by subcutaneous administration. Venous blood samples were collected at time −15, 0, 15, 30, 45 and 60 minutes prior to and after LH administration. The concentrations of the following hormones were measured before LH administration and repeated at each time point of assessment during the LH stimulation test: estradiol (E2), SHBG, T, free T, dehydroepiandrosterone sulphate (DHEAS), Androstenedione (A), and 17 α hydroxyprogesterone (17α-OHP).

Ten women were randomly assigned to receive metformin 500 mg, three times daily and nine women to receive placebo three times daily for the following 2 days. Randomization was accomplished by using a random number table. Metformin and placebo were provided by Kolassa & Merz GmBH (Vienna, Austria) in numbered identical blister packs of six tablets. The subjects were instructed to take one tablet in the morning, mid-day and evening after a meal and not to alter their usual eating habits, physical activity, or lifestyle during the study. A copy of the randomization code was stored in a sealed envelope in the patient’s health record for emergency situations. The randomization code was not broken until the last patient completed all observations. Subjects and investigators were blinded to the treatment allocation throughout the study.

The subjects returned on day 4 and the LH-stimulation test was repeated, as described above.

All study participants were then given metformin 500 mg twice daily for 1 week, followed by 500 mg three times daily to complete twelve weeks of therapy. Patients were advised to use barrier contraception if fertility was not desired and were carefully instructed to stop taking the drug immediately on confirmation of pregnancy. During this follow-up period, each subject returned for weekly clinical assessment including anthropometric measures, evaluation of serum progesterone (level > 5 μg/l confirmed ovulation), and documentation of menstrual bleeding. Repeat hormonal evaluation was performed on cycle day 2 through 7 following a spontaneous menstrual bleed, every 4 to 5 weeks in the absence of menstrual bleeding, and in all women after 12 weeks of metformin use (at the conclusion of the study). After twelve weeks of metformin use, the 3-hour 75 g oral glucose tolerance test with every 15 minute blood assessments for glucose and insulin was repeated, as described above.

Adverse events were recorded throughout the study by direct questioning and by subject self-report.

### Laboratory measures and assays

BMI was calculated using the formula BMI = kg/m^2^.

Glucose was measured with the GOD-PAP-method (glucose oxidase) with a Roche/Hitachi 904 or 917 analyzer. Intra assay-coefficient of variation (CV) was 0.9%, inter assay CV 1.8%. Insulin was measured using the ECLIA method with a Coat-A-Count Insulin In-vitro Diagnostic Test Kit (Diagnostic Products Corporation, USA). The intra assay CV was 1.9%, inter assay CV 2.6%. Crossreactivity with proinsulin was reported as 0.05%.

LH, FSH, E2, DHEAS, SHBG, A, and 17-OHP concentrations were measured with electrochemiluminescence (ECLIA) using commercially available kits (Diagnostic Products Corporation, USA) and analyzed in a Siemens Immulite 2000 Immunoassay system.

T and fT were measured using a radioimmunoassay (RIA) method with a Coat-a-count In- vitro Diagnostic test Kit (Diagnostic Products corporation, USA) in a Siemens Immulite 2000 Immunoassay system. The sensitivity of theses assays were 0.04 μg/L and 0.015 ng/dL, respectively. The intra-assay and inter-assay CV for the range of values pertinent to our patients was less than 10%.

The Free Androgen Index (FAI) was calculated as follows: FAI = total testosterone (nmol/l) × 100/SHBG (nmol/l).

The serial values obtained during the 3-h OGTT were used to calculate the area under the curve (AUC) for both insulin and glucose, using the trapezoidal method, as previously described [19]. IR was defined with an AUC of Insulin exceeding 12,000, as previously described [20]. In a similar fashion, an AUC response over 60 minutes was calculated for each of the serially measured hormones concentrations following LH stimulation.

### Statistical analyses

Descriptive statistics were used for continuous data obtained at each visit. The results are presented as means ± SEM, unless otherwise indicated.

Differences between groups were evaluated using non-parametric tests including the Mann–Whitney U-Test. Within-group differences and repeated measures were evaluated using the paired t-test, Wilcoxon-Test and the Friedman Test.

Data analysis was performed using SPSS 18.0 for Windows (Chicago, IL). P < 0.05 was considered statistically significant.

Sample size was calculated for a within-group treatment effect. Specifically, expecting a decline in T from a mean of 0.6 μg/L to 0.4 μg/L (upper limit of normal in our laboratory) after the 2 days of Metformin and setting an alpha of 0–05, Power (1-Beta) of 0.80, we needed 10 subjects in the treatment arm.

## Results

At the start of the study, all subjects were in the early follicular phase of the cycle, as confirmed by the measurement of FSH, LH, E2 and P at baseline. The baseline anthropometric, metabolic, and hormonal characteristics of the metformin and placebo groups are shown in Table 1.

**Table 1:** **Baseline characteristics of the study groups**

	Metformin	Placebo
*No. of subjects*	10	9
*Age (yrs)*	26.10 ± 4.25	27.89 ± 4.83
*Weight (kg)*	78.97 ± 21,86	95.26 ± 21.64
*BMI (kg/m2)*	29.90 ± 7.41	32.15 ± 6.10
*No. with oligomenorrhea (%)*	10 (100%)	8 (89%)
*No. with PCO on ultrasound (%)*	8 (80%)	7 (78%)
*Total testosterone (μg/l)*	0.58 ± 0.24	0.66 ± 0.36
*SHBG (nmol/l)*	35.60 ± 24.09	39.02 ± 33.30
*Free testosterone (pg/ml)*	1.77 ± 0.99	1.92 ± 0.91
*Androstendione (μg/l)*	3.65 ± 1.47	3.61 ± 0.91
*17-OHP (μg/l)*	1.35 ± 0.37	1.22 ± 0.49
*Free androgen Index*	7.78 ± 4.96	7.92 ± 3.26
*Estradiol (ng/l)*	36.56 ± 14.06	48.00 ± 20.77
*DHEAS (mg/l)*	2.50 ± 0.97	1.84 ± 0.79
*Fasting glucose (mg/dl)*	81.80 ± 6.03	86.89 ± 6.57
*AUC glucose*	18409.70 ± 2059.30	17987.67 ± 2264.72
*(mg/dl x 180 min)*		
*Fasting insulin (μIU/ml)*	11.06 ± 6.38	18.04 ± 10.66
*AUC insulin*	12754.40 ± 8060.54	16671 ± 12663.37
*(μUI/ml x 180 min)*		
No. with IR* (%)	3 (30%)	5 (56%)

Data are given as mean ± SEM unless otherwise noted.

*IR was defined as AUC insulin > 12,000 μUI/ml x 180 min.

At baseline, insulin resistance, defined as an area under the curve of insulin greater than 12,000 μUI/ml × 180 min on the oral glucose tolerance test, was seen in 8 of the 19 subjects, 3 out of 10 of those who subsequently received Metformin and 5 out of 9 of those in the placebo group.

The differences in the baseline (pre-LH administration) hormone concentrations of E, SHBG, T, free T, DHEAS, A, 17α-OHP were calculated between day 1 and day 4, following Metformin or placebo treatment. These results are shown in Table 2 under Static Parameters. There was no change of any of these hormones from day 1 to day 4 within each study group and likewise no difference between study groups.

**Table 2:** **Change (∆) in hormone parameters from study day 1 to day 4, following 2 days of Metformin or placebo treatment**

Static parameters	Metformin	***p***	Placebo	***p***	***p***
		Within group		Within group	Between group
∆ *T (ug/l)*	−0.071 ± 0.147	0.16	0.109 ± 0.306	0.32	0.13
∆ *SHBG (nmol/l)*	−1.430 ± 7.456	0.56	−3.056 ± 5.787	0.15	0.61
∆ *FAI*	−0.640 ± 2,187	0.93	2.311 ± 3.742	0.10	0.05
∆ *DHEAS (mg/l)*	0.153 ± 0.385	0.24	−0.004 ± 0.240	0.96	0.31
∆ *A (μg/l)*	0.00 ± 0.158	0.35	−0.278 ± 0.151	0.97	0.22
∆ *17-OHP (μg/l)*	0.490 ± 0.872	0.59	−0.106 ± 0.114	0.38	0.30
∆ *Free T (pg/ml)*	0.00 ± 0.158	1.0	−0.278 ± 0.151	0.10	0.22
∆ *E2 (ng/l)*	4.899 ± 4.942	0.35	−2.429 ± 4.117	0.58	0.27
***Dynamic parameters***					
∆ *AUC Testosterone*	−8.100 ± 2.188	**0.005**	4.524 ± 4.69	0.36	**0.02**
∆ *AUC SHBG*	−112.725 ± 143.358	0.45	−320.333 ± 119.331	**0.028**	0.11
∆ *AUC DHEAS*	10,501 ± 26,467	0.24	1,508 ± 21,924	0.84	0.45
∆ *AUC A*	−25.275 ± 13.524	0.09	−3.083 ± 16.652	0.86	0.45
∆ *AUC 17-OHP*	1.568 ± 4.698	0.75	−8.449 ± 6.412	0.22	0.40
∆ *AUC Free T*	−26.325 ± 8.058	**0.01**	−18.167 ± 9.014	0.08	0.45
∆ *AUC E2*	273.214 ± 374.027	0.49	−247.500 ± 288.740	0.42	0.46

Statistically significant differences are shown in boldface.

We then evaluated the dynamics of these hormones following LH-administration before and following metfor-min or placebo administration. We again calculated both within- as well as between-group differences. As shown in Table 2, under Dynamic Parameters, there was a significant decrease in the stimulated AUC of T as well as stimulated AUC of free T within the Metformin group only. On between group evaluations, only the difference in the AUC of T statistically differed between the two groups. Namely, following LH-administration, women who received two days of Metformin treatment showed on average a 8.100 ± 2.188 ug*75 min/l *lower* AUC T following LH administration, compared to an average *rise* of 4.524 ± 4.69 ug*75 min/l in the AUC T in women receiving placebo (p = 0.02). In both groups, the LH-stimulated AUC SHBG declined following 2 days of Metformin treatment; in the placebo group, this decline was statistically significant. Nonetheless, there was no difference in the response of SHBG to LH-stimulation between the two groups. With a decline in SHBG, we would expect a rise in T, which was slight and not statistically significant in the control group. On the other hand, the substantial decline in AUC T in the Metformin group occurred despite the slight (and not statistically significant) decline in AUC SHBG.

### Follow-up period

All 19 women agreed to take 1500 mg daily of Metformin in the open-label follow period of the study. One patient became pregnant in the second month of the follow-up period, dropped out of the study, and was thus excluded from further analyses.

During the 12 weeks of Metformin treatment in the 18 women, we were able to document 9 ovulatory cycles (serum Progesterone >5 μg/l) in the first 4 weeks, and 8 ovulatory cycles in each of the following 4-week time intervals.

There was no statistically significant change in weight or BMI after the 12 weeks of follow-up. We were able to repeat the 3 hour oral GTT at the conclusion of the 12 weeks of metformin in 16 of the study participants. There was no change from baseline in fasting insulin or fasting glucose, nor in the calculated AUC glucose and AUC insulin following as part of the OGTT. Likewise, no change in the measured metabolic parameters (total Cholesterol, LDL, HDL, triglycerides) was observed from baseline to following the 12 weeks of metformin administration.

We compared hormone parameters at the start of the study to those at the completion of the study, after 12 weeks of metformin treatment. There was a statistically significant decrease in mean T from 0.63 ± 0.71 μg/l to 0.44 ± 0.45 μg/l, (p = 0.038) and a slight rise in mean DHEAS from 2.23 ± 0.22 mg/l to 2.55 ± 0.28 mg/l (p = 0.029). No change in SHBG, free T, or FAI was seen.

We relied on patient self-report for medication compliance, with all women reporting consistent use at the regular study visits. Side effects were limited to gastrointestinal complaints (nausea, diarrhea), reported by 4 women but none of these women discontinued the medication due to these side effects.

## Discussion

In this randomized controlled study, we found that a short, 2-day course of metformin attenuated significantly the LH-induced testosterone concentration in women with PCOS. SHBG did not increase during this time period (appeared in fact to decline) which excludes the possibility that the T effect was secondary to changes in SHBG.

Our findings therefore suggest that there is a direct effect of metformin on androgen secretion and/or production at the ovarian level, independent of its insulin-sensitizing effects. These observations are consistent with those of Mansfield et al. [9], who reported that in vitro production of androgens by theca cells can be reduced by the addition of metformin.

Previous clinical studies have shown that a beneficial effect on hyperandrogenism appears as early as within a few weeks of treatment with metformin, but several months seem to be required to see the drug’s effects in improving hyperinsulinemia [21, 22]. In the present study, we provide preliminary evidence that the drug’s effects on decreasing androgen production may be even prompter than that, perhaps within as short as a few days.

Although the main aim of this study was to evaluate the immediate effects of metformin on hormonal and metabolic parameters, we did treat all (with the exception of one patient who became pregnant during the study period) participants with 1500 mg metformin daily for a total of 12 weeks in an open-label follow-up. We found no change in body weight and no improvement in insulin sensitivity following three months of treatment, as evidenced by the results of the repeated OGTT at study conclusion. There was likewise no improvement in metabolic parameters, such as LDL, HDL or triglycerides following this relatively short course of treatment. Nonetheless, mean serum T levels did significantly decrease over the 3-month period, consistent with similar findings in a study we previously conducted [23]. The changes in SHBG levels observed in this study over the initial two days are difficult to explain. As they do not exhibit the expected inverse relationship with T levels, they do support our view of a direct effect of metformin on ovarian androgen secretion.

Although this was a prospective, randomized double-blinded controlled trial, the study has a few minor limitations. We did not perform pill counts nor require diaries to confirm subject compliance with the medications. We did, however, see the participants on a weekly basis during the follow-up phase and were reassured that the women were compliant. Since the majority of our subjects (15 out of 19) had hyperandrogenemia, we cannot be certain of the generalizability of our findings to normo-androgenemic women with PCOS who may not demonstrate a comparably large change in testosterone response following short term metformin administration. Although this study included a relatively small number of subjects, we based our sample size calculation on showing within-group differences with each subjects acting as her own control.

## Conclusions

Our data suggest that the effects of metformin on ovarian androgen metabolism in women with PCOS do not require protracted drug exposure and are not secondary to improvement/normalization in insulin sensitivity. Rather, our data imply that there may be a direct effect of the drug at the ovarian level. Thus, the beneficial mechanism of action of metformin in improving hyperandrogenemia in women with PCOS may be more complicated than at first believed, and independent of its insulin actions. Future clinical studies with larger number of subjects are warranted to evaluate these effects more closely and to test their therapeutic implications.
